# Pregnancy and Birth Outcomes during the Early Months of the COVID-19 Pandemic: The MOACC-19 Cohort

**DOI:** 10.3390/ijerph182010931

**Published:** 2021-10-18

**Authors:** Marta Rodríguez-Díaz, Jéssica Alonso-Molero, María J. Cabero-Perez, Javier Llorca, Trinidad Dierssen-Sotos, Inés Gómez-Acebo, The MOACC-19 Group

**Affiliations:** 1 Faculty of Medicine, Universidad de Cantabria, 39011 Santander, Spain; marta.rodriguezd@alumnos.unican.es (M.R.-D.); alonsomoleroj@gmail.com (J.A.-M.); mariajesuscabero@gmail.com (M.J.C.-P.); llorcaj@unican.es (J.L.); 2 IDIVAL Instituto de Investigación Sanitaria Valdecilla, 39011 Santander, Spain; 3 Hospital Universitario Marqués de Valdecilla, 39008 Santander, Spain; 4 CIBER Epidemiología y Salud Pública (CIBERESP), 28029 Madrid, Spain

**Keywords:** SARS-CoV-2 infection, COVID-19, pregnancy, gestational hypertension, placental abruption

## Abstract

The new coronavirus, SARS-CoV-2, is devastating for specific groups of patients, but currently there is not enough information concerning its effects on pregnant women. The purpose of this study is to identify the impact of SARS-CoV-2 infection on pregnancy and the consequences that it could cause. We studied a cohort of pregnant ladies who were tested for SARS-CoV-2 infection by RT-PCR and classified as infected or not infected. The recruitment was carried out in the HUMV hospital, a third-level hospital located in Santander, northern Spain. It started on 23 March 2020 and ended on 14 October 2020. Data from our cohort were compared to another cohort recruited in 2018 at the same hospital. We found that gestational hypertension, placental abruptio, and home exposure to an infected person, among other variables, could be associated with SARS-CoV-2 infection. In conclusion, we consider pregnant women a high-risk group of patients towards a possible SARS-CoV-2 infection, especially those who present with conditions such as gestational hypertension or obesity; moreover, we think that SARS-CoV-2 infection could increase the possibilities of having an abruptio placentae, although this result was found in only a few women, so it requires further confirmation.

## 1. Introduction

In 2019, a new coronavirus emerged in the Hubei province in China, called SARS-CoV-2. Quickly, the virus spread around the world, causing millions of deaths and creating a global health alert [1,2].

SARS-CoV-2 is currently known to be devastating for specific groups of patients, particularly those with previous cardiovascular diseases or elderly patients [3,4]. There is also much information on the consequences of this virus in risk groups such as obese, hypertensive, or diabetic people [5,6]. However, there is not enough information about its effect on another possible risk group, pregnant women [7,8]. Evidence is pointing towards pregnant women as a vulnerable collective based on their higher risk of severe complication of respiratory infections [4]. The reason for this is that they experience a succession of changes in their bodies that implies more susceptibility to respiratory diseases, due to relative suppression of the immune system [9] and some dynamic adjustments, including diaphragmatic raise, respiratory mucosal edema, and augmented oxygen demand, which imply a troublesome adaptation to reduced oxygen levels [10]. All of these changes may facilitate severe outcomes, making future mothers more susceptible to fatal COVID-19 responses, such as a cytokine storm [11,12] and even admission to an ICU (intensive care unit) [8,13]. Furthermore, pregnant women could suffer other comorbidities, which obscures the prognosis of the infected SARS-CoV-2 mother even more.

Some reports have indicated that pregnancy conditions such as gestational hypertension [14], placental abruption [15], or high BMI [16] could determine an increased risk of SARS-CoV-2 infection and the presence of specific SARS-CoV-2 symptoms [4,17]. In this way, arterial hypertension has been demonstrated as one of the major contributors to developing a fatal clinical course of COVID-19 in the general population [6]; in the pregnant collective, where hypertensive disorders are frequent, one of the most prevalent disorders is gestational hypertension, which could constitute a substantial risk factor for COVID-19 disease [18]. Gestational hypertension on its own could be devastating, causing pre-eclampsia, which can lead to placental abruption, abortion, or even death of the mother due to multiple organ failure [19,20]. 

Another risk factor of COVID-19 is obesity. The existing relationship between severe outcomes of COVID-19 and obesity in the general population has been described [21,22], and it is a potential issue for SARS-CoV-2 infection in the gravida collective [16,23]. A hypothesis is that obesity affects lung function in two ways, mechanically resulting in an affected respiratory dynamic as well as inflammation, since there is a state of increased pro-inflammatory cytokines [23,24]. Pregnancy also affects ventilation dynamics due to pressure from the increased uterus [23]. However, gravida patients must be treated with caution, as they can present the same disorders as general patients, but also, specific gravida pathology. An example is the appearance of abruptio placentae, another severe pregnancy complication, which could have a relationship with SARS-CoV-2 infection too considering the presence of protein ACE2 (angiotensin converting enzyme 2) in placental tissues, as a result of SARS-CoV-2 local damage [15]. 

All these factors (hypertensive disorders, obesity, or placental abruption) in the context of COVID-19 could deteriorate a pregnancy, leading to a need for a C-section rather than a natural birth, in order to protect both mother and child [25]. Eutocic birth is the first option of delivery in almost every pregnancy, as it benefits mother and child, but when pregnancy complications are present, a C-section is chosen instead [26]. SARS-CoV-2 infection has been recognized as a clinical criterion to perform a C-section instead of a natural birth, especially if pregnant women present with COVID-19 symptoms [25].

Apart from SARS-CoV-2 risk factors, it is important to mention other virus characteristics such as symptoms, manner of transmission, and immune defense. SARS-CoV-2 infection causes a wide spectrum of symptoms, from asymptomatic patients to acute distress syndrome [27]; a fever and cough are the most common symptoms [1]. However, the symptoms that are particularly distinct to COVID-19 are anosmia and ageusia [28,29]. These are observed both in general patients and in pregnant patients [30,31]. Another important issue concerning SARS-CoV-2 is the manner of transmission, which could occur through small respiratory droplets or direct contact. Respiratory droplets can travel 1.5 meters, which is the reason why social distancing is a fundamental method of prevention. Direct contact with infested areas and fecal–oral transmission are the reasons why hand hygiene is an effective way to stop transmission [32]. The immune system plays an important role against SARS-CoV-2 infection, and during the course of pregnancy the immune system is commonly stimulated by vaccines. This is done in order to activate humoral immunity of the mother, increasing the levels of the mother´s immunoglobulin G antibodies that pass through placental tissues, conferring protection to the newborn [33]. Some vaccines can provide cross-immunization to other micro-organisms [34]. To the best of our knowledge, no other studies have reported if the vaccination state of the mother could affect the infection course of SARS-CoV-2.

The purpose of this article is to describe how COVID-19 has affected pregnancy and which factors could contribute to facilitate SARS-CoV-2 infection in future mothers.

## 2. Materials and Methods

The Mother and Child COVID-19 (MOACC-19) cohort is described elsewhere [35]. The cohort recruitment was carried out in the HUMV hospital, a third-level hospital located in Santander, the capital of Cantabria, a region in the north of Spain. This is where all births in Cantabria took place from March 2020 to June 2020 due to the unstable situation created by the COVID-19 pandemic. Starting on 23 March, all pregnant women admitted for/in labor were tested for SARS-CoV-2 infection by RT-PCR.

The official recruitment started on the 26 of May and was divided into three different subcohorts based on birth date.

The first subcohort includes women who had given birth from the 23 of March to the 25 of May 2020. All had undergone a RT-PCR test just before delivery and were called via telephone and asked if they wanted to be part of the study. It is necessary to highlight their exposure time occurred during their third trimester of pregnancy.

The second subcohort incorporates data from women who gave birth between 26 May and 14 October 2020. In contrast to the first subcohort, they were asked to participate during their stay in the hospital. Their exposure to SARS-CoV-2 could have happened during their second trimester, the same time the first wave in Spain took place, or, on the contrary, exposure could have occurred in their third trimester of pregnancy, as most of the women gave birth before the second wave hit Spain.

Finally, the third subcohort compiles data from pregnant women who were asked to enter the study at their 12-week prenatal revision. The ones who agreed were RT-PCR tested immediately. These women experienced the first pandemic wave during the first trimester of their pregnancy, then they experienced the stabilization of cases during the second trimester and finally an increase in cases during the second pandemic wave in the third trimester of their pregnancy.

### 2.1. Data Collection

Women admitted to the study started by filling in a survey in front of a qualified interviewer. Questions were directed to collect various forms of data: sociodemographic, medical history, obstetric history, COVID-19 symptoms, and exposure history to COVID-19. In addition, both obstetric and medical history were to be confirmed and completed using medical archives. The symptoms that were quantified were fever, chills, tiredness, sore throat, cough, shortness of breath, headache, nausea, vomiting or diarrhea, and loss of taste or smell. We focused on two parameters, presence of taste or smell loss, as well as on the number of symptoms.

### 2.2. Biological Determinations

SARS-CoV-2 detection by RT-PCR was carried out with nasopharyngeal swabs provided to every woman using the HUMV clinical protocol. Those from subcohort 1 and 2 had been tested before giving birth, and those in subcohort 3 were tested the moment they agreed to participate in the study (12 weeks of pregnancy). Moreover, serological studies were done as a part of the study, taking blood samples from every pregnant woman and analyzing IgM or IgG SARS-CoV-2 presence, with ELISA using the IrsiCaixa published protocol [36].

If any test turned positive, RT-PCR and ELISA analysis were to conducted on the woman’s partner.

### 2.3. Comparison Group

Data from our cohort were compared with another cohort of 969 neonates and their mothers recruited in 2018 at the same hospital. It was collected from January to August 2018, gathering data from medical records and from interviews conducted on the mothers.

### 2.4. Statistical Analysis

Statistical comparisons were calculated by the *X*^2^ test or independent samples Student’s *t*-test.

### 2.5. Ethical Considerations

The study was designed according to Declaration of Helsinki (Fortaleza’s last renovation) and regulation 2016/679 of the European Union. All pregnant women had to sign two written informed consents, one for themselves and one for their neonates. They agreed and signed before entering the study.

## 3. Results

The study sample is described in Table 1. Out of the 620 pregnant women in our study, there were 591 negative (95.3%) and 29 positive (4.7%) pregnant women. In both groups the average age of the mother was 33 years, near to 90% were European, and almost half of the sample of both groups were average weight. Of the women, 77% had vocational training or a university education, and 75% were active workers and left work around the 24th week of pregnancy. The predominant fertilization type was natural, 91% in negative mothers and 89% in positive mothers. Only 86 were single mothers (14%) and 9 of them were infected with the novel coronavirus (10.5%). Type of delivery did not show any significant difference either, with natural birth being the most common mode of delivery in both groups (76% in negative mothers and 59% in positive mothers). We observed a trend for more C-section deliveries in positive mothers (26% vs. 18%), but the difference was not significant. Regarding pathology in pregnancy, gestational diabetes (GD) and GD with insulin were similarly distributed in both groups. Additionally, chronic hypertension, pre-eclampsia, placenta previa, threat of miscarriage, metrorrhagia, prelabor rupture of membranes, stillbirth, or chorioamnionitis, among others, did not show any significant difference between infected and non-infected mothers.

Table 1 also shows some significant results, such as gestational weight gain, where we have seen a major rise in percentage in non-infected mothers, who gained an average of 12 kg in comparison to infected mothers, who gained around 10 kg. Table 2 shows the association between pregnancy characteristics and infection by SARS-CoV-2. These results show that being a single mother was associated with an increased risk of SARS-CoV-2 infection by approximately 198% compared to non-single mothers (OR: 2.98, 95% CI: 1.31–6.78). The BMI of pregnant women before pregnancy did not appear to be significantly different between both groups; obesity, in comparison to average weight, was not associated with a risk factor in our cohort (OR: 1.12; 95% CI: 0.37–3.47). The information of Table 1, divided by subcohort, is attached in the Supplementary Material (Table S1).

Table 2 shows that eutocic birth was the most prevalent type of delivery in both groups, followed by C-section, and that assisted birth was the least common mode of delivery. Our results did not find any significant difference between delivery type.

Table 3 shows the number of pregnancies associated with SARS-CoV-2 infection; in both groups the majority of mothers were having their first child. A second pregnancy was associated with a 71% lower risk of infection, compared to mothers who had given birth for the first time (OR: 0.29; 95% CI: 0.09–0.89).

Having gestational hypertension was also more prevalent in SARS-CoV-2-infected mothers, 7%, in contrast to 4% of negative mothers (Table 1). Table 4 shows the association between pregnancy-associated pathology and infection by SARS-CoV-2. Gestational hypertension was associated with a five-fold increased rate of SARS-CoV-2 (OR: 5.09; 95% CI: 1.6–16.05) and placental abruption showed that it could be associated with a 21-fold increased rate of SARS-CoV-2 (OR: 20.93; 95% CI: 1.28–343.3). These results should be considered with caution due to the small size sample. We did not find any relationship between SARS-CoV-2 infection state and pre-eclampsia apparition. The vaccination state of the mother was also studied, considering four variables: non-vaccinated, pertussis-vaccinated, influenza-vaccinated, and pertussis- and influenza-vaccinated during pregnancy. The results (Table 2) show that non-vaccinated mothers were associated with 73% less risk of SARS-CoV-2 infection (OR: 0.27; 95% CI: 0.12–0.62). The information of Table 2, divided by subcohort, is attached in the Supplementary Material (Table S2).

Table 1 shows that COVID-19 RT-PCR results from the women’s partners were statistically different, being positive 0.89% of the time when the mothers were negative and 25.93% of the time when the mothers were positive. Table 5 displays information about the association between social exposure and infection by SARS-CoV-2. Epidemiological exposure history has a significant impact, showing that 8 out of 21 mothers who experienced home exposure were infected with SARS-CoV-2; therefore, home exposure, after having a COVID-19-diagnosed family member, was associated with a 44-fold increased rate of SARS-CoV-2 infection (OR: 43.73; 95% CI: 13.12–145.01). We also considered if contact at home with a relative who suffered flu-like symptoms affected the rates in some way; this was significant too, resulting in a three-fold increased rate of SARS-CoV-2 infection (OR 2.96; 95% CI: 1.07–8.21).

Table 6 shows COVID-19-like symptoms reported by pregnant women according to their SARS-CoV-2 infectious state. We focused on two parameters: presence of taste or smell loss as well as the number of symptoms. The symptoms that were quantified were: fever, chills, tiredness, sore throat, cough, shortness of breath, headache, nausea, vomiting or diarrhea, and loss of taste or smell. It was seen that loss of smell and taste was highly related to COVID-19, resulting in a seven-fold increase in the rate of infection (OR: 6.96; 95% CI: 2.11–22.9), and patients who had six or more symptoms, numbered above, were associated with a five-fold increased risk of infection (OR: 4.94; 95% CI: 0.99–24.78). The information of Table 6, divided by subcohort, is attached in the Supplementary Material (Table S3).

## 4. Discussion

### 4.1. Gestational Arterial Hypertension—SARS-CoV-2-Infected Women

According to our results, gestational hypertension was associated with a five-fold higher risk of SARS-CoV-2 infection in comparison to non-hypertensive pregnancy. Other studies have seen this association as well [8,37]; for example, Zambrano et al. postulate that gravida patients under cardiovascular risk, including hypertension, were more likely to experience severe SARS-CoV-2 infection than other same-aged pregnant women [8]. Syeda et al. said that the hypertensive population have a higher risk of developing severe COVID-19, and they assume that gestational hypertension puts pregnant women at risk of critical disease, although they did not find any significant results [37]. Bandara et al. observed that the course of gestational hypertension includes dysfunction in RAAM (renin–angiotensin–aldosterone mechanism) activity, and if SARS-CoV-2 infection is associated, it could significantly deteriorate pregnancy [38]. Kayem et al. described that hypertensive disorders in gravida patients are a risk factor of acute COVID-19, particularly if they have an associated higher risk of SARS-CoV-2 infection due to pre-eclampsia [39]. In contrast, other studies have considered this relationship, but they could not find any association [16,40]. Grechukhina et al. thought that it could be attributable to the small number of participants in their study [16]. Schwartz et al. have seen an association between hypertensive disorders and SARS-CoV-2-infected women; however, they did not consider it a risk factor for SARS-CoV-2 infection or vertical transmission [17]. Sinnot et al. have seen an increased rate of hypertensive disorders among SARS-CoV-2-infected mothers, but they did not observe any link with poor outcomes [41]. The relationship between gestational arterial hypertension and SARS-CoV-2 can be explained by the aetiopathogenesis of the virus, given that ACE2 (angiotensin-converting enzyme 2) conforms the gateway for SARS-CoV-2 and that this union activates the RAAM [38,42]. Physiologically, ACE2 performs a vital role in maintaining regular levels of blood pressure in gravida patients due to its function in the RAAM. However, when pregnant women suffer gestational hypertension or pre-eclampsia they experience a progressive dysfunction of RAAM, which makes us think that SARS-CoV-2 infection could lead to severe outcomes in these groups of pregnant patients, given that the RAAM would be affected in two ways: in one way due to dysfunction related to the pathogenesis of gestational hypertension and, in another way, due to SARS-CoV-2 infection [15,38,43]. Xuan et al. studied RAAS and ACE2 behavior during pregnancy and concluded that women with gestational hypertension had lower levels of other proteins that conform RAAS (activated renin and angiotensin 2), but they did not find changes in ACE2 levels between hypertensive and non-hypertensive pregnant women. Hence, these findings suggest that dysregulation of RAAS in the gestational hypertension context could be triggered by other non-known factors, which could not affect the ACE2 enzyme. Thus, if RAAS is disrupted in the gestational hypertension context, not affecting ACE2, it could get worse if a concomitant SARS-CoV-2 infection appears, because the virus will bond with the ACE2 enzyme, affecting the RAAM dysfunction even more [44]. In addition, some authors reflect that hypertensive pregnant woman should be considered a part of the “high-risk” group patients who could undergo a fatal response [10,45,46]. However, some papers posit that the state of pregnancy creates an anti-inflammatory profile that prevents them from developing a cytokine storm [12,47,48]. This hypothesis is based on the fact that during pregnancy the immune system is influenced by progesterone and human chorionic gonadotropin hormone, which are present at high levels during pregnancy, and both are involved in diminishing TNFα, which blocks the lymphocyte Th1 pathway [47]. Hence, the Th2 pathway is the predominant pathway during pregnancy; in addition, pregnant women have high levels of interleukin-10 (IL-10), which protects the mother from COVID-19 [48]. Thus, as a result of high IL-10 levels and the activation of the Th2 pathway, the inflammatory response is reduced, leading to a reduction in severe inflammatory outcomes of COVID-19 in the pregnant population compared to the normal population [48].

In view of these conflicting results, further investigation is needed. In the meantime, based on our results, we think that hypertensive disorders during pregnancy put future mothers at risk, and they should be monitored closely to prevent fatal outcomes. It should be mentioned that the authors did not discuss the possibility of complications derived from SARS-CoV-2 infection in pregnant women. Our study, however, was designed with a prospective follow-up, which will allow us to adequately identify complications that may appear after being infected by SARS-CoV-2.

### 4.2. Placental Abruption

Our data display that placental abruption is associated with a 21-fold increased risk of SARS-CoV-2 infection. Other studies have also observed this relationship [15,38]; for example, Bandara et al. considered that there is a possible relationship between SARS-CoV-2 infection and placental disfunction [38]. In the same way, Hosier et al. have published a case report of one pregnant woman affected by COVID-19, pre-eclampsia, and placental abruption, whose placenta was analyzed; they observed placental invasion of SARS-CoV-2 [15]. On the other hand, Li et al. noticed an increased number of preterm births as a consequence of gestational complications, including placental abruption; however, they do not think the apparition of pregnancy complications are a result of SARS-CoV-2 infection [25]. In the same way, McDonnel et al. did not see any difference between maternal outcomes during the pandemic period compared to maternal outcomes during the years 2018 and 2019 [40]. A possible explanation for the relationship between SARS-CoV-2 pathogenesis during pregnancy and placental abruption could be the physiological presence of ACE2 in placental tissues [42], which explains why anatomopathological studies of placentas from SARS-CoV-2-infected women had this virus present in syncytiotrophoblast cells [12,15]. This hypothesis is supported by Li et al., given that pregnancy pathologies linked to COVID-19 are typical of late stages in pregnancy, and they found that the presence of ACE2 in placental cells increases as pregnancy evolved, showing very low expression at the beginning of the pregnancy and rising from the 24th week of pregnancy until the end [43]. In addition, this hypothesis is also supported by the presence of the virus on placental tissues, which has been proven and could mean an activation of immune mechanisms, creating an inflammatory response to the placenta [49].

Placental defense against infection is performed by mechanical barriers, immune cell positioning, and immunological pathways. All of them are necessary to keep placental integrity during the course of SARS-CoV-2 infection and protect against poor placental outcomes. This implies that in cases where these defenses are damaged, placental infection and its poor consequences are more frequent. Kreis et al. said that the disruption of these lines could be occurring in mothers who suffer pre-eclampsia, hypertension, obesity, and other diseases, making these groups more vulnerable to SARS-CoV-2 infection [14]. However, this hypothesis is not the only one; another way the placenta could be affected is by vascular thrombosis in placental vessels. Kreis et al. have also reported deposits of fibrin and vascular malperfusion in placental studies, which they have linked to placental insufficiency [14]. In the same line, Turan et al. have reported 14 cases of placental dysfunction in SARS-CoV-2-infected woman, who also had other outcomes, such as miscarriage or bleeding, resulting in thrombotic phenomena [50]. However, some authors did not find any anatomopathological difference between placentas from infected mothers and placentas from a control group [51]. 

Therefore, there is evidence concerning a possible link between placental abruptio and COVID-19, but it will be necessary to conduct more investigations in this field to clear up the etiopathogenesis.

### 4.3. Birth and COVID-19

We did not find any significant results concerning the type of birth of SARS-CoV-2-infected women, and others did not find any either [25]. However, the C-section rate in 2018 was 23% of all assisted births in Cantabria [52], and in our cohort 27% of SARS-CoV-2-infected women went through a C-section. Hence, in absolute numbers, the rate of C-section was higher. We think that our non-significant results could be explained by the small sample, considering that many hospitals experienced an increase in the C-section rate during the first wave of the COVID-19 pandemic [53]. An example of this was reported by McDonnell et al., who found a positive relationship between periods of higher death rates in Ireland and the number of C-sections [40]. This association was especially strong at the beginning of 2020, when data on infected mothers were insufficient to clarify which type of birth was more secure for mothers, neonates, and obstetricians [27]. Globally, a state of uncertainty around professionals’ security was established, and the development of a eutocic birth was considered a high-risk situation for transmission, which led to the preference of performing a C-section rather than a natural birth on COVID-19-infected women [54]. On the other hand, some infected mothers presented serious comorbidities, such as hypertensive disorders or diabetes, which made it impossible for a natural birth to occur. In this case, SARS-CoV-2 infection may be a confusion factor in the relationship between C-section and mothers’ comorbidities [4]. Others have associated the performance of a C-section to poor mother outcomes [55]. In conclusion, choosing a C-section above natural delivery could be justified by the fear of transmission during eutocic birth and for the infected mother’s own safety [50,54].

### 4.4. Symptoms of COVID-19 in Pregnancy

Our patients showed that having six or more symptoms of COVID-19 multiplies the association with SARS-CoV-2 infection by five. We have considered the following COVID-19 symptoms: fever, chills, tiredness, sore throat, cough, shortness of breath, headache, nausea, vomiting or diarrhea, and loss of taste or smell. Specifically, anosmia or ageusia were associated with a seven-fold increased risk of SARS-CoV-2 infection. We did not find any other reports where symptoms were counted per gravida, showing only that the two most prevalent symptoms among the pregnancy collective were cough and fever, followed by dyspnea [3,4,47,56,57]. According to our results, Dubey et al. published a metanalysis where the data suggest a correlation between severity of symptoms and adverse pregnancy outcomes—a greater number of symptoms led to an increased risk of poor pregnancy outcomes. However, they explain that case report studies show results from more severe COVID-19 patients, and therefore results could be confusing [56]. Berhan et al. observed that pregnant women infected with SARS-CoV-2, who had an underlying autoimmune condition, experienced more severe symptoms during the first trimester of pregnancy than others during the second or third semester [48]. In contrast, Chen et al. found that among infected pregnant women there was a significant number of asymptomatic women [10]. Gao et al. have concluded that pregnant women are less susceptible to present any symptom than the rest of population [58]. On the other hand, Cosma et al. have also studied anosmia and ageusia in a group of COVID-19-positive pregnant patients, but they did not find any difference between SARS-CoV-2-infected and non-infected pregnant women in their first trimester of pregnancy [59]. Still, these findings are in contrast to evidence concerning why pregnant ladies are at more risk of respiratory disease, since some pregnancy modifications take place in the immune system, resulting in a relative suppression of it [9]. Besides, they experience some dynamic adjustments, including diaphragmatic raise, respiratory mucosal oedema, and augmented oxygen demand, which facilitate the apparition of symptoms [10].

### 4.5. Home Transmission

Our results support the previous information, showing that intrafamilial home contact with someone who had been diagnosed of COVID-19 results in a three-fold increased risk of SARS-CoV-2 infection. This result is interesting given that most reports based on pregnant, infected ladies do not distinguish between home transmission or an epidemiological history of exposure. For example, Li et al., in a case–control study on a pregnant lady infected by SARS-CoV-2, could not report any contact with someone infected. This study took samples from pregnant ladies between 23 January and 29 February of 2020 in the Hubei province, China [25]. Another study performed by Zhu and colleagues showed results of nine pregnant ladies who went into labor from 20 January to 5 February of 2020. Eight of them lived in Wuhan, where the coronavirus pandemic started, and just one of them could report close contact with her husband, who was infected by SARS-CoV-2 [60]. Almost every study made in Wuhan, China included pregnant ladies who had an epidemiological exposure history [17], and some systematic reviews found that the majority of patients also had an exposure history [61]. A case report had a similar home transmission result to ours, but the sample was just one pregnant lady [15]. Hence, all these results are in accordance with our results, which show a significant relationship between SARS-CoV-2 infection and contact at home with an infected person.

### 4.6. Vaccines and SARS-CoV-2-Infected Women

Our results implied that non-vaccinated mothers had a lower risk of infection and that mothers vaccinated with influenza vaccines had a higher risk of infection than pertussis-vaccinated mothers. However, there is not enough literature that studies this possible association, which made it hard to compare our results to others. We only found a report by Li et al. where they published an analysis of a long list of risk factors for COVID-19. They studied how immunization with DTP and a measles vaccine was related to COVID-19 cases, deaths, and case fatality rates. Measles immunization appeared to be related to higher risk of COVID-19 deaths and DTP immunization to an increase in the case fatality rate [62]. The importance of studying this variable falls into two of the principal vaccines indicated during pregnancy, which are for influenza and pertussis [33]. Inactivated influenza vaccines have demonstrated a substantial decrease in adverse effects of respiratory disease secondary to the influenza virus. This was observed in mothers and their children below 6 months old [63]. The aim of the pertussis vaccine is to reduce neonatal deaths by passive protection of the infant, showing around 90% protection against *B. pertussis* infection [64]. We believe in a potential relationship between vaccination state of the mother and SARS-CoV-2 infection, but more lines of investigation about this should be necessary.

## 5. Conclusions

In conclusion, we consider pregnant women a high-risk group of patients towards a possible SARS-CoV-2 infection, especially those who present conditions such as gestational hypertension or obesity. Both conditions have demonstrated a relationship with a fatal response of the mother in the context of COVID-19 disease, provoking a deterioration in the natural course of pregnancy. What is known until now points out that general hypertensive disorders are a detrimental condition in general patients, and the study of the pregnancy collective during the pandemic allows us to think they belong to those hypertensive risk patients. Moreover, SARS-CoV-2 infection seems to increase the possibilities of having an abruptio placentae. Hence, infected pregnant women should be intensely followed up, checking mothers’ health by monitoring blood pressure and explaining the alarm signs of abruptio placentae (gestational bleeding) to them in order to prevent catastrophic responses. However, more lines of investigation in this area should be performed to clarify other potential risks of pregnant women.

## Figures and Tables

**Table 1:** Main characteristics of women included in study.

Variable		Whole Cohort (*n* = 620)		
		COVID-19		
		Negative	Positive	*p*
**Variable cohort profile**				
Subcohort 1		253 (42.81)	13 (44.83)	0.830
Subcohort 2		338 (57.19)	16 (55.17)	
**Age, mean ± SD**		33.59 (0.21)	32.72 (0.94)	0.370
**Age**				
<25		32 (5.43)	1 (3.45)	0.0524
25–29		77 (13.07)	9 (31.03)	
30–34		208 (35.31)	5 (17.24)	
35–39		203 (34.47)	11 (37.93)	
>40		69 (11.71)	3 (10.34)	
Pre-pregnancy BMI *				
Low weight (<20)		83 (14.24)	3 (10.71)	0.692
Average weight (20–25)		287 (49.23)	16 (57.14)	
Overweight (25–30)		149 (25.56)	5 (17.86)	
Obesity (>30)		64 (10.98)	4 (14.29)	
**Nationality**				
European		519 (89.02)	25 (86.21)	0.138
African		8 (1.37)	2 (6.90)	
Asian		4 (0.69)	0 (0.00)	
Latino-American		52 (8.92)	2 (6.90)	
**Education level**				
Primary		79 (13.50)	4 (13.79)	0.987
Secondary		50 (8.55)	2 (6.90)	
Vocational training		188 (32.14)	9 (31.03)	
University		268 (45.81)	14 (48.28)	
**Working status**				
Unemployed/non-active worker		136 (23.29)	9 (31.03)	0.547
Employed		441 (75.51)	20 (68.97)	
Student		7 (1.20)	0 (0.00)	
**Gestational age at which work was left, mean ± SD**		24.30 (0.48)	24.35 (2.19)	0.981
**Fertilization type**				
Natural		532 (91.57)	25 (89.29)	0.638
Artificial insemination		7 (1.20)	1 (3.57)	
In vitro fertilization (own ovules)		31 (5.34)	1 (3.57)	
In vitro fertilization (donated ovules)		11 (1.89)	1 (3.57)	
**Pregestational BMI, mean ± SD**		24.35 (0.16)	24.86 (0.83)	0.548
**Gestational weight gain, mean ± SD**		12.17 (0.21)	10.20 (0.96)	0.045
**Gestational age at positive result to infection of SARS-CoV-2, mean ± SD**			39.31 (1.07)	
**Smoker in pregnancy**	No	504 (85.86)	27 (93.10)	0.269
	Yes	83 (14.14)	2 (6.90)	
**Alcohol consumption in pregnancy**	No	559 (95.23)	29 (100.00)	0.229
	Yes	28 (4.77)	0 (0.00)	
**Parity (including current delivery)**				
1		232 (39.52)	15 (51.72)	0.0455
2		213 (36.29)	4 (13.79)	
≥3		142 (24.19)	10 (34.48)	
**Type of delivery**				
Eutocic		439 (75.82)	17 (58.62)	0.087
Instrumental		36 (6.22)	4 (13.79)	
Caesarean section		104 (17.96)	8 (27.59)	
**COVID-19 RT-PCR (partner)**				
Negative		555 (99.11)	20 (74.07)	<0.001
Positive		5 (0.89)	7 (25.93)	
**Gestational age at delivery according to vaccination status**				
Pertursis, mean ± SD		39.24 (1.44)	39.29 (1.11)	0.935
Influenza, mean ± SD		39.88 (0.83)	39.5 (0.71)	0.581
Pertursis and influenza, mean ± SD		39.18 (2.40)	39.32 (1.16)	0.8
**Pathology in pregnancy**				
Gestational diabetes	No	548 (92.72)	26 (89.66)	0.538
	Yes	43 (7.28)	3 (10.34)	
Gestational diabetes with insulin	No	567 (95.94)	27 (93.10)	0.457
	Yes	24 (4.06)	2 (6.90)	
Gestational hypertension	No	573 (96.95)	25 (86.21)	0.002
	Yes	18 (3.05)	4 (13.79)	
Chronic hypertension	No	586 (99.15)	29 (100.00)	0.619
	Yes	5 (0.85)	0 (0.00)	
Pre-eclampsia	No	569 (96.28)	27 (93.10)	0.387
	Yes	22 (3.72)	2 (6.90)	
Placenta previa	No	585 (98.98)	29 (100.00)	0.586
	Yes	6 (1.02)	0 (0.00)	
Fetal malformations	No	903 (99.56)	35 (100.00)	0.694
	Yes	4 (0.44)	0 (0.00)	
Placental abruptio	No	566 (95.77)	28 (96.55)	0.838
	Yes	25 (4.23)	1 (3.45)	
Threat of miscarriage	No	885 (97.57)	34 (97.14)	0.871
	Yes	22 (2.43)	1 (2.86)	
Metrorrhagia (second half of pregnancy)	No	587 (99.32)	28 (96.55)	0.103
	Yes	4 (0.68)	1 (3.45)	
Prelabor rupture of membranes	No	588 (99.49)	29 (100.00)	0.701
	Yes	3 (0.51)	0 (0.00)	
Stillbirth	No	591 (100.00)	29 (100.00)	-
Threat of premature delivery	No	893 (98.46)	35 (100.00)	0.459
	Yes	14 (1.54)	0 (0.00)	
Chorioamnionitis	No	590 (99.83)	29 (100.00)	0.825
	Yes	1 (0.17)	0 (0.00)	
Number of habitants at home	2	69 (12.15)	5 (17.86)	0.635
	3	235 (41.37)	10 (35.71)	
	4 or more	264 (46.48)	13 (46.43)	
Number of usual visitors	No	268 (46.29)	10 (34.48)	0.213
	Yes	311 (53.71)	19 (65.52)	
Contact at home with a positive person	No	574 (99.14)	21 (72.41)	<0.001
	Yes	5 (0.86)	8 (27.59)	
Contact with positive family or friends	No	533 (92.06)	27 (93.10)	0.838
	Yes	46 (7.94)	2 (6.90)	
Contact with someone with flu-like symptoms at home	No	541 (93.44)	24 (82.76)	0.029
	Yes	38 (6.56)	5 (17.24)	
Contact with family or friends with flu-like symptoms	No	520 (89.81)	29 (100.00)	0.070
	Yes	59 (10.19)	0 (0.00)	
Symptoms	No symptoms	356 (61.49)	16 (55.17)	0.207
	One–two symptoms	172 (29.71)	9 (31.03)	
	Three–five symptoms	42 (7.25)	2 (6.90)	
	Six symptoms or more	9 (1.55)	2 (6.90)	

* Body mass index.

**Table 2:** Association between pregnancy characteristics and infection by SARS-CoV-2.

Variable	SARS-CoV-2-Infected/Non-Infected	OR (95% CI)	*p*
**Single parent**			
No	20/510	1 (reference)	-
Yes	9/77	2.98 (1.31–6.78)	0.009
**BMI**			
Low weight	3/83	0.65 (0.18–2.28)	0.499
Average weight	16/287	1 (reference)	.
Overweight	5/149	0.60 (0.22–1.68)	0.331
Obesity	4/64	1.12 (0.37–3.47)	0.843
**Weight gain in pregnancy**			
0–8.9 kg	8/111	1.32 (0.52–3.33)	0.554
9.0–12.9 kg	12/220	1 (reference)	-
13.0–15.9 kg	2/113	0.32 (0.07–1.47)	0.145
16 kg or more	6/137	0.80 (0.29–2.19)	0.668
**Vaccines in pregnancy**			
None	1/14	1.40 (0.18–11.22)	0.75
Pertussis	7/192	0.72 (0.30–1.73)	0.46
Influenza	2/8	4.90 (0.97–24.72)	0.05
Pertussis and influenza	19/373	1 (reference)	-
**Birth type**			
Eutocic	17/439	1 (reference)	-
Instrumentally assisted	4/36	2.86 (0.92–8.98)	0.07
Caesarean section	8/104	1.98 (0.83–4.83)	0.12

**Table 3:** Demographic characteristics and reproductive history. Their relationships with infection by SARS-CoV-2.

Number of Pregnancies	SARS-CoV-2-Infected/Non-Infected	OR (95% CI)	*p*
1	15/232	1 (reference)	-
2	4/213	0.29 (0.09–0.89)	0.03
≥3	10/142	1.09 (0.48–2.49)	0.84

**Table 4:** Association between pregnancy-associated pathology and infection by SARS-CoV-2.

Pathology	SARS-CoV-2-Infected/Non-Infected	OR (95% CI)	*p*
**Gestational arterial hypertension**	4/18	5.09 (1.60–16.05)	0.006
**Pre-eclampsia**	2/22	1.92 (0.43–8.57)	0.39
**Placental abruption**	1/1	20.93 (1.28–343.3)	0.03

**Table 5:** Association between social exposure and infection by SARS-CoV-2.

Variable	SARS-CoV-2-Infected/Non-Infected	OR (95% CI)	*p*
**Contact at home with a relative diagnosed with COVID-19**			
No	21/574	1 (reference)	-
Yes	8/5	43.73 (13.12–145.01)	<0.001
**Contact at home with a relative suffering flu-like symptoms**			
No	24/541	1 (reference)	-
Yes	5/38	2.96 (1.07–8.21)	0.04

**Table 6:** COVID-19-like symptoms reported by pregnant women according to their SARS-CoV-2 infection status.

Symptoms	SARS-CoV-2-Infected/Non-Infected	OR (95% CI)	*p*
**Loss of taste or smell**	4/13	6.96 (2.11–22.9)	0.001
**Number of symptoms**			
**0 symptoms**	16/356	1 (reference)	-
**1–2 symptoms**	9/172	1.16 (0.50–2.69)	0.72
**3–5 symptoms**	2/42	1.06 (0.24–4.77)	0.94
**≥6 symptoms**	2/9	4.94 (0.99–24.78)	0.05

## Data Availability

The data presented in this study are available on request from the corresponding author. The data are not publicly available due to patients’ privacy.

## Supplementary Materials

The following are available online at https://www.mdpi.com/article/10.3390/ijerph182010931/s1, Table S1: Main characteristics of women included in study by subcohort, Table S2: Association between pregnancy characteristics and infection by SARS-CoV-2 by subcohort, Table S3: COVID-19-like symptoms reported by pregnant women according to their SARS-CoV-2 infection status by subcohort.
