# Supplementary material for: Pregnancy and Birth Outcomes during the Early Months of the COVID-19 Pandemic: The MOACC-19 Cohort
Source: Int J Environ Res Public Health. 2021 Oct 18;18(20):10931. doi: 10.3390/ijerph182010931 (PMC8535818; doi:10.3390/ijerph182010931)
Supplement: Supplementary file 1 [file ijerph-18-10931-s001.zip › ijerph-1366593-supplementary.pdf]

SUPPLEMENTARY MATERIAL

Table S1. Main characteristics of women included in study by subcohort

	Subcohort 1 (n = 266)			Subcohort 2 (n = 354)		
	COVID-19			COVID-19		
	Negative	Positive	p	Negative	Positive	p
**Age, mean±SD**	33.91(0.30)	33.46(1.34)	0.745	33.35(0.29)	32.13(1.31)	0.363
**Age**						
<25	10(3.95)	0(0.00)	0.752	22(6.55)	1(6.25)	0.042
25–29	31(12.25)	3(23.08)		46(13.69)	6(37.50)	
30–34	87(34.39)	4(30.77)		121(36.01)	1(6.25)	
35–39	94(37.15)	5(38.46)		109(32.44)	6(37.50)	
>40	31(12.25)	1(7.69)		38(11.31)	2(12.50)	
**Pre-pregnancy BMI (body mass index) ***						
Low weight (<20)	36(14.34)	1(7.69)	0.369	47(14.16)	2(13.33)	0.870
Average Weight (20-25)	121(48.21)	7(53.85)		166(50.00)	9(60.00)	
Overweight (25-30)	69(27.49)	2(15.38)		80(24.10)	3(20.00)	
Obesity (>30)	25(9.96)	3(23.08)		39(11.75)	1(6.67)	
**Nationality**						
European	228(90.48)	13(100.00)	0.715	291(87.92)	12(75.00)	0.043
African	2(0.79)	0(0.00)		6(1.81)	2(12.50)	
Asian	1(0.40)	0(0.00)		3(0.91)	0(0.00)	
Latino-American	21(8.33)	0(0.00)		31(9.37)	2(12.50)	
**Education level**						
Primary	32(12.65)	1(7.69)	0.681	47(14.16)	3(18.75)	0.926
Secondary	18(7.11)	0(0.00)		32(9.64)	2(12.50)	
Vocational training	94(37.15)	5(38.46)		94(28.31)	4(25.00)	
University	109(43.08)	7(53.85)		159(47.89)	7(43.75)	
**Working status**						
Unemployed/non active worker	53(21.12)	3(23.08)	.915	83(24.92)	6(37.50)	0.495
Employed	195(77.69)	10(76.92)		246(73.87)	10(62.50)	
Student	3(1.20)	0(0.00)		4(1.20)	0(0.00)	
**Gestational age leave work, mean±SD**	25.44(0.70)	22.90(2.96)	0.404	23.43(0.66)	25.80(3.19)	0.467
**Gestational age at infection of SARS-2, mean±SD**	39.09(2.68)	38.92(0.95)	0.829	39.31(1.49)	39.63(1.09)	0.408
**Fertilisation type**						
Natural	233(92.83)	12(92.31)	0.939	299(90.61)	13(86.67)	0.259
Artificial insemination	2(0.80)	0(0.00)		5(1.52)	1(6.67)	
In vitro fertilisation (own ovules)	13(5.18)	1(7.69)		18(5.45)	0(0.00)	
In vitro fertilisation (donated ovules)	3(1.20)	0(0.00)		8(2.42)	1(6.67)	
**Pregestational BMI, mean±SD**	24.24(0.30)	26.31(1.34)	0.132	24.33(0.28)	23.78(1.33)	0.687
**Gestational weight gain, mean±SD**	11.99(5.11)	9.42(9.70)	0.096	12.37(0.27)	10.87(1.26)	0.243

**Smoker in pregnancy**	No	224(88.54)	11(84.62)	0.667	280(83.83)	16(100.00)	0.080
	Yes	29(11.46)	2(15.38)		54(16.17)	0(0.00)	
**Alcohol consumption in pregnancy**	No	240(94.86)	13(100.00)	0.402	319(95.51)	16(100.00)	0.386
	Yes	13(5.14)	0(0.00)		15(4.49)	0(0.00)	
**Parity (including current delivery)**							
1		97(38.34)	6(46.15)	0.139	135(40.42)	9(56.25)	0.218
2		103(40.71)	2(15.38)		110(32.93)	2(12.50)	
≥3		53(20.95)	5(38.46)		89(26.65)	5(31.25)	
**Type of delivery**							
Eutocic		188(74.90)	7(53.85)	0.220	251(76.52)	10(62.50)	0.373
Instrumental		17(6.77)	2(15.38)		19(5.79)	2(12.50)	
Caesarean rate		46(18.33)	4(30.77)		58(17.68)	4(25.00)	
**COVID-19 RT-PCR (partner)**							
Negative		237(100.00)	10(90.91)	<0.001	318(98.45)	10(62.50)	<0.001
Positive		0(0.00)	1(9.09)		5(1.55)	6(37.50)	
**Pathology in pregnancy**							
Gestational diabetes	No	235(92.89)	13(100.00)	0.319	313(92.60)	13(81.25)	0.1
	Yes	18(7.11)	0(0.00)		25(7.40)	3(18.75)	
Gestational diabetes with insulin	No	242(95.65)	11(84.62)	0.072	325(96.15)	16(100.00)	0.424
	Yes	11(4.35)	2(15.38)		13(3.85)	0(0.00)	
Gestational hypertension	No	246(97.23)	11(84.62)	0.014	327(96.75)	14(87.50)	0.055
	Yes	7(2.77)	2(15.38)		11(3.25)	2(12.50)	
Chronic hypertension	No	251(99.21)	13(100.00)	0.748	335(99.11)	16(100.00)	0.705
	Yes	2(0.79)	0(0.00)		3(0.89)	0(0.00)	
Pre-eclampsia	No	244(96.44)	13(100.00)	0.489	325(96.15)	14(87.50)	0.093
	Yes	9(3.56)	0(0.00)		13(3.85)	2(12.50)	
Placenta previa	No	250(98.81)	13(100.00)	0.693	335(99.11)	16(100.00)	0.705
	Yes	3(1.19)	0(0.00)		3(0.89)	0(0.00)	
Placental abruptio	No	240(94.86)	12(92.31)	0.687	326(96.45)	16(100.00)	0.443
	Yes	13(5.14)	1(7.69)		12(3.55)	0(0.00)	
Metrorrhagia (second half pregnancy)	No	251(99.21)	12(92.31)	0.021	336(99.41)	16(100.00)	0.758
	Yes	2(0.79)	1(7.69)		2(0.59)	0(0.00)	
Prelabour rupture of membranes	No	252(99.60)	13(100.00)	0.820	336(99.41)	16(100.00)	0.758
	Yes	1(0.40)	0(0.00)		2(0.59)	0(0.00)	
Stillbirth	No	253(100.00)	13(100.00)	-	338(100.00)	16(100.00)	-
Chorioamnionitis	No	253(100.00)	13(100.00)	-	337(99.70)	16(100.00)	0.828
	Yes				1(0.30)	0(0.00)	
Number of habitants at home	2	3(1.21)	1(7.69)	0.151	66(20.63)	4(26.67)	0.843
	3	103(41.53)	4(30.77)		132(41.25)	6(40.00)	
	4 or more	142(57.26)	8(61.54)		122(38.13)	5(33.33)	
Number of usual visitors	No	137(55.02)	4(30.77)	0.087	131(39.70)	6(37.50)	0.861
	Yes	112(44.98)	9(69.23)		199(60.30)	10(62.50)	
Contact at home with a positive person	No	247(99.20)	11(84.62)	<0.001	327(99.09)	10(62.50)	<0.001
	Yes	2(0.80)	2(15.38)		3(0.91)	6(37.50)	
Contact with positive family or friends	No	229(91.97)	13(100.00)	0.288	304(92.12)	14(87.50)	0.508
	Yes	20(8.03)	0(0.00)		26(7.88)	2(12.50)	
Contact with someone with flu-like symptoms at home	No	234(93.98)	12(92.31)	0.807	307(93.03)	12(75.00)	0.009
	Yes	15(6.02)	1(7.69)		23(6.97)	4(25.00)	

Contact with family or friends with flu-like symptoms	No	220(88.35)	13(100.00)	0.192	300(90.91)	16(100.00)	0.207
	Yes	29(11.65)	0(0.00)		30(9.09)	0(0.00)	
Symptoms	No symptoms	145(58.23)	9(69.23)	0.656	211(63.94)	7(43.75)	0.001
	1-2 symptoms	78(31.33)	4(30.77)		94(28.48)	5(31.25)	
	3-5 symptoms	20(8.03)	0(0.00)		22(6.67)	2(12.50)	
	6 symptoms or more	6(2.41)	0(0.00)		3(0.91)	2(12.50)	

Table S2. Association between pregnancy characteristics and infection by SARS-CoV-2 by subcohort

**Variable**	**Subcohort 1**			**Subcohort2**		
	**SARS-CoV-2 infected/non infected**	**OR (95% CI)**	**p**	**SARS-CoV-2 infected/non infected**	**OR (95% CI)**	**p**
**Single parent**						
No	8/203	1 (reference)	-	12/307	1 (reference)	-
Yes	5/50	2.54 (0.80-8.09)	0.11	4/27	3.79 (1.14-12.56)	0.029
**BMI**						
Low weight	1/36	0.48 (0.06-4.03)	0.499	2/47	0.78 (0.16-3.76)	0.762
Average weight	7/121	1 (reference)	-	9/166	1 (reference)	-
Overweight	2/69	0.50 (0.10-2.48)	0.397	3/80	0.69 (0.18-2.62)	0.588
Obesity	3/25	2.07 (0.5-8.58)	0.314	1/39	0.47 (0.06-3.84)	0.484
**Weight gain in pregnancy**						
0 – 8.9 kg	4/55	1.31 (0.34-5.08)	0.697	4/56	1.33 (0.37-4.71)	0.662
9.0 – 12.9 kg	5/90	1 (reference)	-	7/130	1 (reference)	-
13.0 – 15.9 kg	0/49	-	-	2/64	0.58 (0.12-2.87)	0.505
16 kg or more	4/55	1.31 (0.34-5.08)	0.697	2/82	0.45 (0.09-2.23)	0.331
**Vaccines in pregnancy**						
None	0/4	-	-	1/10	2.3 (0.26-20.56)	0.75
Pertussis	0/33	-	-	7/159	1.01 (0.35-2.95)	0.02
Influenza	1/4	4.4 (0.46-42.63)	0.199	1/4	5.75(0.57-58.41)	1.48
Pertussis and influenza	12/212	1 (reference)	-	7/161	1 (reference)	-
**Birth type**						
Eutocic	7/188	1 (reference)	-	10/251	1 (reference)	-
Instrumentally assisted	2/17	3.16 (0.6-16.42)	0.171	2/19	2.64 (0.54-12.93)	0.231
Caesarean section	4/46	2.33 (0.66-8.31)	0.191	4/58	1.73 (0.52-5.71)	0.368

Table S3. Covid-19-like symptoms reported by pregnant women, according to their SARS-CoV-2 infection status by subcohort:

Symptoms	Subcohort 1			Subcohort 2		
	SARS-CoV-2 infected/non infected	OR (95% CI)	p	SARS-CoV-2 infected/non infected	OR (95% CI)	p
**Loss of taste or smell**	2/9	4.85 (0.93-25.17)	0.06	2/4	11.64 (1.96-69.02)	0.007
**Number of symptoms**						
**0 symptoms**	9/145	1 (reference)	-	7/211	1 (reference)	-
**1-2 symptoms**	4/78	0.82 (0.25-2.77)	0.76	5/94	1.6 (0.5-5.18)	0.43
**3-5 symptoms**	0/20	-	-	2/22	2.74 (0.54-14.01)	0.23
**≥6 symptoms**	0/6	-	-	2/3	20.09 (2.88-140.0)	0.002
